# Validation of a Single RGB-D Camera for Gait Assessment of Polyneuropathy Patients

**DOI:** 10.3390/s19224929

**Published:** 2019-11-12

**Authors:** Maria do Carmo Vilas-Boas, Ana Patrícia Rocha, Hugo Miguel Pereira Choupina, Márcio Neves Cardoso, José Maria Fernandes, Teresa Coelho, João Paulo Silva Cunha

**Affiliations:** 1 Institute for Systems Engineering and Computers – Technology and Science (INESC TEC), and Faculty of Engineering (FEUP), University of Porto, 4200-391 Porto, Portugal; mcarmo.vilasboas@gmail.com (M.d.C.V.-B.); hugo.choupina@gmail.com (H.M.P.C.); 2 Unidade Corino de Andrade, Hospital Santo António, Centro Hospitalar Universitário do Porto, E. P. E., 4099-001 Porto, Portugal; marcio.neves.cardoso@gmail.com (M.N.C.); tcoelho@netcabo.pt (T.C.); 3 Institute of Electronics and Informatics Engineering of Aveiro (IEETA), and Department of Electronics, Telecommunications and Informatics, University of Aveiro, 3810-193 Aveiro, Portugal; aprocha@ua.pt (A.P.R.); jfernan@ua.pt (J.M.F.)

**Keywords:** gait, 3-D motion analysis, neuropathy, RGB-D camera, system validity

## Abstract

Motion analysis systems based on a single markerless RGB-D camera are more suitable for clinical practice than multi-camera marker-based reference systems. Nevertheless, the validity of RGB-D cameras for motor function assessment in some diseases affecting gait, such as Transthyretin Familial Amyloid Polyneuropathy (TTR-FAP), is yet to be investigated. In this study, the agreement between the Kinect v2 and a reference system for obtaining spatiotemporal and kinematic gait parameters was evaluated in the context of TTR-FAP. 3-D body joint data provided by both systems were acquired from ten TTR-FAP symptomatic patients, while performing ten gait trials. For each gait cycle, we computed several spatiotemporal and kinematic gait parameters. We then determined, for each parameter, the Bland Altman’s bias and 95% limits of agreement, as well as the Pearson’s and concordance correlation coefficients, between systems. The obtained results show that an affordable, portable and non-invasive system based on an RGB-D camera can accurately obtain most of the studied gait parameters (excellent or good agreement for eleven spatiotemporal and one kinematic). This system can bring more objectivity to motor function assessment of polyneuropathy patients, potentially contributing to an improvement of TTR-FAP treatment and understanding, with great benefits to the patients’ quality of life.

## 1. Introduction

Human body movement offers physicians important clues on the progression of certain disabling disorders affecting movement, such as Transthyretin Familial Amyloid Polyneuropathy (TTR-FAP). TTR-FAP is a highly incapacitating and inherited transthyretin amyloidosis, which has a wide range of clinical manifestations [1]. The V30M mutation is usually characterized by a neuropathic phenotype, which begins by affecting the sensory and autonomic fibers and later the motor fibers, thus affecting mobility and progressively constraining the patients’ quality of life [1]. The disease has been classified into three stages: I—sensory polyneuropathy, II—progressive walking disability, and III—wheelchair bound or bedridden [2].

Currently, TTR-FAP has no cure and, if left untreated, patients have a survival rate of 2 to 15 years after onset [3]. Even when treated, patients continue to have disease progression [4,5]. Nevertheless, an early diagnosis improves the chances of a successful treatment [1], which is essential to maintain the patients’ quality of life over time.

In neurological disorders, a quantitative motion analysis tool would bring more objectivity to motor function assessment, helping with the medical decisions regarding diagnosis and/or treatment. However, quantitative motion analysis is rarely used in clinical scenarios, with the mobility level being most commonly assessed in a subjective way (e.g., visual observation) [6]. This is probably due to the lack of solutions that are suitable for use in clinical settings (i.e., accurate, inexpensive, portable, not intrusive for the patients, and simple to setup and use). 

The quantified assessment of gait characteristics in patients affected by TTR-FAP is still an exploratory subject. We have previously studied the possibility of using a low-cost, portable and minimally invasive RGB-D camera (Microsoft Kinect v2) for supporting the diagnosis of this disease based on gait analysis [7], but did not validate it against a reference tool. To the best of our knowledge, this type of study has never been carried out before for this disease.

Therefore, the present study intends to assess the use of a system based on a single RBG-D camera, namely the Kinect v2, for obtaining gait parameters in the context of TTR-FAP, which can be used to support the clinical gait assessment of patients. In contrast with our preliminary studies involving Parkinson’s disease and TTR-FAP patients [7,8], where the Kinect was not validated against a reference system, this study evaluates the agreement between the Kinect v2 and a reference Qualisys system for each considered parameter.

3-D body joint data provided by both systems were acquired from ten TTR-FAP patients while they performed ten gait trials. Instead of exploring the time series for the whole gait trial as in [9], we computed 23 spatiotemporal and kinematic parameters for each performed gait cycle. These gait parameters include ten more spatiotemporal and eight kinematic parameters that were not considered in our previous work on automated gait analysis [10]. To compute some of the parameters, it was further necessary to detect a new type of gait event (toe off).

## 2. Background and Related Work

The reference systems for motion capture are systems that are able to accurately capture and track the movements of a given subject, by relying on multiple cameras and several retroreflective markers placed on the body [11]. These systems have been extensively used in the past for different applications, including clinical applications [12]. Although the reference systems are very accurate, they have several disadvantages, such as being expensive, intrusive, not very portable, and requiring specific knowledge (for calibration, etc.) [13], making their use mostly restricted to laboratories and, hence, not suitable for use in a clinical environment.

RGB-D cameras are a more appropriate alternative for clinical practice, due to their low-cost, portability and minimal intrusiveness [14]. A single RGB-D camera, such as the Kinect, is able to track several body joints in 3-D, based on depth data obtained relying on infrared light [14], without requiring calibration or the use of markers. Nonetheless, the provided 3-D joint data can be less accurate than those of reference systems [15], being thus important to evaluate its validity for each specific goal (e.g., gait assessment for a given disease).

The Kinect has already been validated in different contexts for both healthy subjects [16,17,18,19,20] and patients with movement impairments, such as the assessment of the maximum walking speed and static posturography in multiple sclerosis [21,22,23] and the assessment of different movements in Parkinson’s Disease [24]. In the case of gait analysis, it was found that the Kinect can be used for obtaining different spatiotemporal gait parameters/measures in healthy populations [9,10,18,19] and patients with multiple sclerosis [21]. However, its use is not recommended for measuring most kinematic gait parameters/measures, in healthy populations [9,19,20]. In the context of Parkinson’s disease, the Kinect was shown to measure timing and gross spatial characteristics in an accurate way, during clinically relevant movements, including walking in the same place [24].

## 3. Materials and Methods

### 3.1. Participants 

We conducted an experiment at LABIOMEP (Porto Biomechanics Laboratory) with the participation of ten TTR-FAP patients (seven male and three female). Their demographic data is presented in Table 1. Patients were clinically recruited and the inclusion criteria were the following: being a V30M mutation carrier, and being followed in external consultation at Corino de Andrade Unit (Centro Hospitalar Universitário do Porto, Porto, Portugal). The only exclusion criteria were the presence of feet injuries, the presence of any other lower extremity disorder or disease that may alter gait kinematics, and the inability to walk independently (no other limits, such as age limit, were imposed). The experiment was approved by the hospital’s Ethics Committee and complies with the Declaration of Helsinki. All subjects signed an informed consent form.

The patients had TTR-FAP and PND (polyneuropathy disability) scores of 1 (4 patients) or 2 (6 patients), with heterogeneous gait when compared to each other. However, all alterations resulted from the sensory-motor polyneuropathy caused by the disease. All patients presented sensory ataxia and steppage gait, with different degrees of instability and movement coordination during stride.

Regarding treatment and diagnosis, four patients had been taking medication (tafamidis) for 10 months, 3 years and 4 months, 3 years and 5 months, and 3 years and 7 months, with 2, 11, 7 and 6 years passed since diagnosis, respectively. Other four patients had been subjected to a liver transplant 6, 8, 12 and 16 years ago, and 8, 13, 17 and 20 years had passed since diagnosis, respectively. The two remaining patients were enrolled in different clinical trials for not responding to tafamidis and had been diagnosed 5 and 6 years ago.

### 3.2. Experimental Setup and Protocol

**Figure 1:**
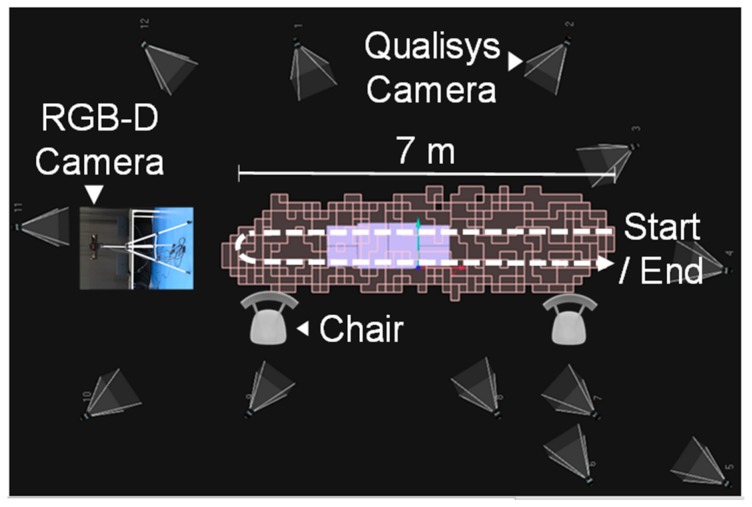
Experimental setup including an RGB-D camera (Kinect v2) mounted on a tripod, 12 infrared cameras of the Qualisys system and two chairs. The Qualisys’ calibrated volume is illustrated by the salmon-colored blocks. The walking path carried out by the subjects is represented by the dashed arrowed line. This figure was adapted from the Qualisys setup image provided by LABIOMEP (Porto Biomechanics Laboratory).

The experimental setup, illustrated in Figure 1, consisted of a reference motion capture system (Qualisys) that included twelve Oqus infrared cameras and 61 retro-reflective markers attached to the patients’ body as indicated in [10], according to the setup typically used in LABIOMEP for full body gait analysis. The setup also comprised a single RGB-D camera (Kinect v2) placed in front of the participant, with a height of 1 m and tilt angle of −5 degrees, to maximize the sensor’s practical depth range. 

The experimental protocol consisted of walking towards and away from the Kinect, at a self-selected pace, for 14 m (turn-around carried out at 1.2 m from the sensor). Each subject repeated this task ten times. To aid the most fragile participants, the setup also included two chairs: one at the beginning and another at the end of the walking path (Figure 1). Patients were free to hold the chair when turning around or at the end of each trial.

For synchronization purposes, an extra marker was dropped on the floor within the field of view of both used systems, before the beginning of each gait trial.

### 3.3. Data Acquisition and Pre-Processing

**Figure 2:**
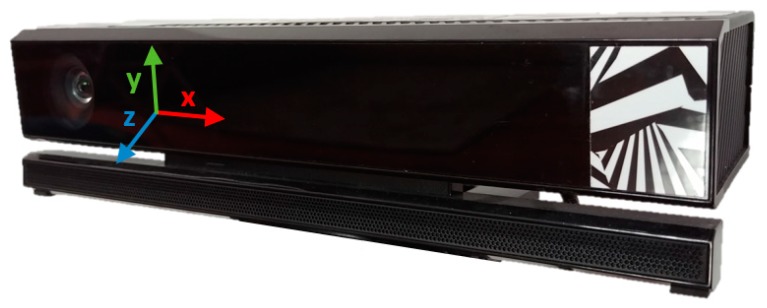
3-D coordinate system of the Kinect v2, located at the center of the infrared sensor [26].

The 3-D position of the Qualisys’ markers was acquired at 200 Hz, with an accuracy of at least 0.6 mm. Data provided by the Kinect—3-D body joint, depth and infrared—were acquired at 30 Hz, using our *KiT* software application [25]. Each body joint frame includes the 3-D position of the twenty-three joints tracked by Kinect (see [10]). The 3-D coordinate system associated with the Kinect v2 is illustrated in Figure 2. The data from both systems were synchronized, and the time intervals corresponding to walking towards the Kinect were automatically selected, using the methods described in [9].

### 3.4. Gait Cycle Phases Detection

The gait cycles and associated phases (stance and swing, single- and double-limb support), performed by each patient, were automatically identified by detecting two types of gait events: Heel strikes and toe offs.

The actual gait events were identified based on the feet vertical velocity computed over Qualisys data, according to the findings of O’Connor et al. [27]. The used signals were processed using a zero-lag low-pass fourth order Butterworth filter with a cutoff frequency of 8 Hz (value chosen based on the signals’ frequency content).

For the RGB-D camera, to decide if a given frame corresponds to a heel strike instant, we relied on the distance between ankles signal and a window with a size of ND1 frames centered at the current frame, as further detailed in [10]. The side (left or right) associated with each detected heel strike was identified relying on the ankles’ velocity signals, considering a window with a size of ND2 frames centered at the corresponding heel strike instant frame [10]. The used signals were previously processed using a moving average filter, considering a window size of NF1 and NF2 frames for heel strike detection and side identification, respectively. The best window sizes were selected using the method indicated in [10].

In the present study, we additionally detected toe offs by finding the maximum of the absolute difference between the angle of the left and right shanks, when considering the frames between the instant corresponding to a heel strike and the following instant when the ankle distance is minimum. The shank angle is defined by the knee joint, ankle joint and the point with knee y-coordinate and ankle x- and z-coordinates. The shank angle signals were previously processed using a moving average filter with a window size of NF3 frames. The value of NF3 was chosen by varying it between 1 and 11 frames (odd integer values) and then choosing the one that led to the best trade-off between sensitivity and mean absolute error for estimating the duration of the different gait cycle phases.

### 3.5. Gait Parameter Computation

For each detected gait cycle, we computed the 23 gait parameters—15 spatiotemporal and 8 kinematic—listed in Table 2 (unless stated, all spatial and kinematic measures were computed in 3-D, i.e., considering all the 3 axes). They include two spatiotemporal parameters usually not explored in gait analysis studies: Total Body Center of Mass (TBCM) sway x- and y-component. These parameters measure the variability of TBCM motion, which can provide useful information regarding the posture and/or balance during gait. This information can be important to help predict fall risk in patients with gait impairments [28].

For Qualisys, the 3-D position of each joint was computed from the 3-D position of the relevant marker or the midpoint between two markers. These data were then processed using a zero-lag low-pass fourth order Butterworth filter with a cutoff frequency of 15 Hz (value chosen according to the signals’ frequency content). 

In the case of Kinect, some of the measures used to compute the gait parameters were also processed using a zero-lag low-pass Butterworth filter. For each measure, we explored filter orders of 2, 4 and 6, as well as cutoff frequencies between 1 and 9 Hz inclusive (integer values). We then elected the pair of values for each gait parameter that led to the best trade-off between the mean absolute error, when comparing Kinect and Qualisys, considering all gait cycles from all subjects.

For each parameter and gait cycle, we discarded the obtained value if the signal used to compute that parameter had at least one outlier. Detailed information on outlier detection is presented in [9].

Finally, we computed the mean value of each parameter per subject. To investigate if the number of considered gait cycles has any influence on the agreement between the Kinect and Qualisys, we varied it between 1 and 10, obtaining the metrics described below. The value of 10 gait cycles corresponds to the maximum number for which all subjects had a value for at least one of the gait parameters. If the number of detected gait cycles for a given subject was higher than the defined amount, the used gait cycles were selected randomly.

All signal processing and analyses described in this and the previous subsection were performed in MATLAB (version R2016b).

### 3.6. Validation of the Kinect v2

The validity of the RGB-D camera (Kinect v2) for obtaining gait parameters was evaluated against the reference motion capture system (Qualisys), by obtaining for each parameter the Bland-Altman’s mean difference and 95% limits of agreement (LoA), as well as the Pearson’s and concordance correlation coefficients and associated 95% confidence interval (CI), between the two systems.

The Pearson’s correlation coefficient (*r*) indicates the relative agreement between two variables, i.e., the strength of their linear relationship [30]. The concordance correlation coefficient (*r*_c_) indicates the absolute agreement between variables, by measuring not only how far each observation deviates from the line fit to the data (precision), but also how far this line deviates from the 45 degree line through the origin (accuracy) [31]. The value of *r* and *r*_c_ ranges between −1 and 1, where −1 means perfect disagreement, 0 corresponds to an independence situation and 1 indicates perfect agreement [32]. Based on the guidelines given by Portney and Watkins [33], we set the correlation thresholds as poor (<0.5), moderate (≥0.5 and <0.75), good (≥0.75 and <0.9) and excellent (≥0.9).

We also detected the presence of fixed and/or proportional bias. Fixed bias corresponds to a constant difference in measurements between devices, and was detected using a one-sample t-test on the differences between systems [34]. There is no fixed bias if the mean difference is indistinguishable from zero (*p*-value ≤ 0.05) [34]. Proportional bias occurs when a system returns values higher or lower than the other system by an amount proportional to the level of the measured variable. It was detected by performing a OLS (ordinary least squares) regression analysis [34], where there is proportional bias if the OLS regression line fitted to the plot of the differences on means has a slope that differs significantly from zero (*p*-value ≤ 0.05).

All the analyses described in this section were performed in the R environment (version 3.5.1) [35]. The *stats* package [35] was used to compute the Pearson’s correlation coefficient and associated 95% CI and *p*-value, as well as for performing the t-test and OLS regression analysis. The concordance correlation coefficient and associated 95% CI were obtained using the *cccrm* package [36].

## 4. Results

Regarding the detection of gait cycles and associated phases, the sizes selected for the used windows were the following: 7 frames for NF1 and ND1, 13 frames for NF2, 9 frames for ND2 and NF3. These window sizes led to a precision of 98.4% and sensitivity of 91.9% when detecting heel strikes, and a perfect identification of the heel strikes’ side (precision and sensitivity of 100%). A sensitivity of 98.1% was achieved for detecting toe offs. The results obtained for all explored window sizes are shown in Tables S1–S3 of the supplementary material.

As for the Kinect measure filtering, the selected values for the Butterworth filter’s parameters are indicated in Table S4 of the supplementary material (complete results in Tables S5 and S6).

**Figure 3:**
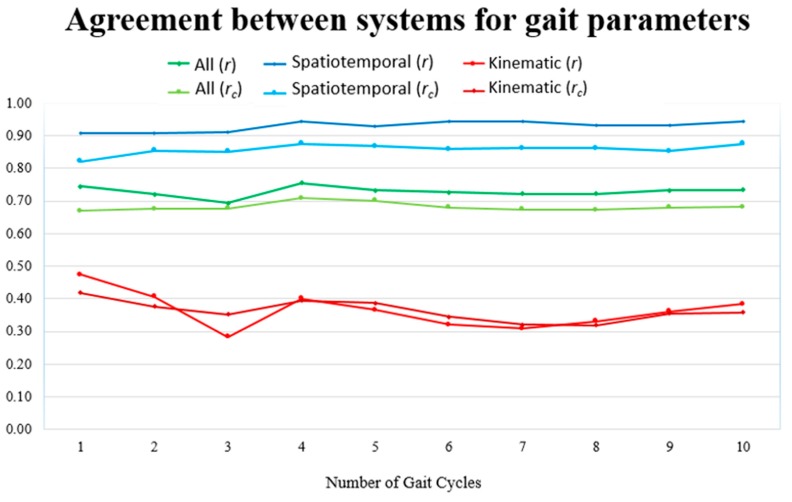
Mean value for Pearson’s and concordance correlation coefficients (*r* and *r_c_*, respectively) for spatiotemporal, kinematic and all gait parameters, versus the number of gait cycles used to compute the parameter value per subject.

When considering the agreement between Kinect and Qualisys, the mean *r* and *r*_c_ values for spatiotemporal, kinematic and all gait parameters, versus the number of gait cycles used for parameter computation, are presented in Figure 3. As can be seen from this figure, the values did not vary considerably when increasing the number of gait cycles from 1 to 10. Nevertheless, the best results in the case of all parameters were achieved when using four gait cycles.

The validation results obtained for each gait parameter, when using four gait cycles per subject, are presented in Table 3. The results include the mean and standard deviation values (Mean ± SD) for Kinect and Qualisys, as well as the Bland Altman’s mean difference (Mean diff) between the two systems and the associated 95% limits of agreement (LoA). The presence of fixed and/or proportional bias is indicated for each parameter by the ˜ and ˆ symbols, respectively. The results also include the Pearson’s and concordance correlation coefficients (*r* and *r*_c_, respectively), and the associated 95% confidence interval (CI). The *r* values that are significant (*p*-value ≤ 0.05) are marked with the * symbol.

## 5. Discussion

When comparing the results obtained in the present study, and the investigation we previously performed with healthy subjects [10], the achieved precision and sensitivity values were overall similar for heel strike detection (precision of 98% vs 99%, and sensitivity of 92% vs 98%) and side identification (precision and sensitivity of 100% vs >99.7%). The biggest difference was observed for the heel strike detection sensitivity (lower for TTR-FAP patients), which was expected due to the presence of gait abnormalities that most likely make the detection of actual heel strikes more difficult. The detection of toe offs was not carried out with healthy subjects. Nevertheless, the sensitivity of 98% obtained with TTR-FAP patients was relatively high, allowing the computation of all considered temporal parameters for almost all detected gait cycles.

The agreement between the Kinect and Qualisys for the obtained gait parameters was overall better for spatiotemporal than kinematic parameters. This finding is in accordance with the results reported in previous studies on the validation of either the Kinect v1 or v2 for gait assessment in healthy populations [18,19,20].

Most spatiotemporal parameters (eleven out of fifteen) presented excellent relative and absolute agreement (*r* ≥ 0.93 and *r*_c_ ≥ 0.92). TBCM sway y-component also had excellent relative agreement (*r* = 0.97), but the absolute agreement was only moderate (*r*_c_ = 0.73). For the remaining spatiotemporal parameters (swing, single support and double support duration), relative agreement was good (0.81 ≤ *r* ≤ 0.89), while absolute agreement was moderate (0.57 ≤ *r*_c_ ≤ 0.68).

The TBCM sway is not usually considered in studies on RGB-D camera validation for gait assessment. However, we have included it in this study because it provides information on the patients’ balance during gait, which may help identify the tendency to lower the body center of mass with the onset of sensory ataxia and therefore contribute to an earlier diagnosis of sensory losses. The results show that the TBCM sway, especially when considering the x-axis, can be extracted from the Kinect data (excellent agreement between systems).

In the case of kinematic parameters, the best results were obtained for the spine shoulder angle and minimum elbow angle, with excellent relative agreement (*r* = 0.9) and moderate or good absolute agreement (*r*_c_ = 0.69 and *r*_c_ = 0.88, respectively). The minimum knee angles had good relative agreement, but the absolute agreement was poor. The good result achieved for the minimum elbow angle is interesting given that parameters related with the elbows’ angle are also usually not included in studies on Kinect’s validity for gait analysis, although they may provide important information on the movement of the subjects’ arms, during gait.

It is also interesting to note that the maximum elbow/knee angles and hip angle range present a mean difference of 0 (zero) degrees, but the relative/absolute agreement is moderate or poor. This happened because, although the mean difference between systems considering all patients is of 0 degrees (rounded, for the hip angle range, as an example, it is –0.4 degrees), the difference for each subject varies between negative and positive values (e.g., between –9 and 8 degrees, in the case of the hip angle range), corresponding to a relatively high mean absolute difference (e.g., 4 degrees for the hip angle range) and low *r* and *r*_c_ values.

Another aspect that is worth noting is the fact that the spine shoulder and spine middle angles (associated with the trunk), and the minimum and maximum knee angle, are considerably different despite their computation relying on the same or similar measures. In the case of the trunk angles, the spine middle angle not only presents a higher mean error, but also a higher upper 95% limit of agreement, leading to worse agreement results. As for the knees, although the minimum knee angle has a higher mean error than the maximum knee angle, the first angle presents only positive errors per patient, while those errors vary between negative and positive values leading to a worse agreement for the second angle.

The poor/different results discussed above, for the kinematic parameters, may be due to something as simple as a reflective marker not being correctly placed or the occurrence of a body part occlusion for some of the patients, which may have influenced the reference measurement. It is also possible that the configuration used for the Kinect (height, tilt angle, and position regarding the walking path), and/or the noise reduction of the 3-D data provided for certain joints, need to be further optimized to improve accuracy in measuring specific angles/parameters.

Fixed bias was detected for 9 out of the 23 parameters, while proportional bias was found for a lower number of parameters (5 out of 23). Both fixed and proportional bias were detected for four parameters. Despite of the presence of fixed and/or proportional bias for ten parameters, they can both be corrected by generating calibration equations, using ordinary least products (OLP) regression analysis [34].

This study’s results cannot be directly compared with those attained in our previous work on Kinect’s validity with healthy subjects [9], since in the previous work we carried out a point-to-point analysis for different measures, while in the present contribution we obtained gait parameters corresponding to the mean/maximum/minimum/range value for some of those measures. Nevertheless, the *r* results obtained with TTR-FAP patients for gait speed (*r* = 1.00), foot and arm swing velocity (*r* = 0.96 and *r* = 0.99), and hip angle range (*r* = 0.30) are in line with our findings in a healthy population regarding full-body velocity (*r* = 0.89), lower-lower-limb and upper limb velocity (*r* = 0.92 and *r* = 0.99), and hip angle (*r* = 0.23), respectively [9]. These results show that several gait parameters/measures can be extracted using the Kinect v2 in people both with and without gait impairments, validating its use for supporting clinical gait assessment in different contexts. Although the Kinect v2 has been discontinued, a new version of this camera (Azure Kinect DK) relying on the same depth sensing approach (time-of-flight) is already available in some countries [37].

In the specific case of clinical scenarios, providing accurate information is very important. However, nowadays the patient assessment is still mainly performed in a qualitative way, using subjective scores [12]. Therefore, we believe that the use of a system based on an RGB-D camera, such as the Kinect, represents a good compromise between obtaining some relevant and useful quantitative information about the disease onset and progression (even though not as complete and accurate as the reference systems, such as the Vicon and Qualisys systems) and not being too expensive, time-consuming, intrusive for the patients, and restricted to specific physical spaces (e.g., motion analysis laboratory). The possibility of performing gait analysis anywhere within the hospital, or even at the patients’ home, allows the medical staff to save time and consequently assess patients’ gait more frequently. Moreover, the information provided by RGB-D camera-based systems, such as the one validated in this study, can be useful for disease diagnosis or disease progression evaluation during patients’ follow-up, despite not being so detailed and precise.

The performed study has some limitations, including the low number of participants and the fact that they were under different treatments, which was mainly due to the difficulty in recruiting patients and evaluating them in a laboratory away from the hospital, since they are subjected to a great amount of examinations in each visit to the hospital center. In addition, the walking pace was self-selected, which may have caused variations from patient to patient, from trial to trial for the same patient or even within the same trial. These limitations can be overcome by recruiting more patients and selecting patients with similar treatments, as well as controlling the pace with sound reference or similar approaches. We are currently acquiring data, using the Kinect v2, from more TTR-FAP patients, which we intend to use in future studies with a larger number of patients.

## 6. Conclusions

In this contribution, we evaluated the validity of an RGB-D camera-based system, against a reference motion capture system, for supporting clinical gait assessment of patients suffering from Transthyretin Familial Amyloid Polyneuropathy (TTR-FAP), a highly disabling condition frequently affecting gait, which is usually assessed through subjective observation. To the best of our knowledge, this is the first study on the validity of an RGB-D camera for gait assessment in TTR-FAP and peripheral neuropathy in general.

Our study showed that this low-cost, portable and minimally intrusive system, based on a single RGB-D camera, is able to obtain most of the fifteen studied spatiotemporal parameters. These parameters include the TBCM sway, which is important for studying the patients’ balance during gait. As for the kinematic parameters, only the elbow angle minimum obtained with our system can be used for gait assessment.

These quantified gait parameters can be used to develop a more objective and fine-grained motor/gait assessment scale, which would be very useful for helping the physicians performing an earlier and better TTR-FAP diagnosis and/or a more optimized treatment during patient follow-up, leading to the improvement of the patients’ quality of life.

As future work, it would be interesting to explore other parameters to identify disease-specific features, as well as verify if an RGB-D camera can be used to distinguish between different disease stages and/or detect fine changes in the gait pattern of each patient during disease progression.

## Figures and Tables

**Table 1:** Characterization of the subjects that participated in the experiment. SD stands for standard deviation.

	Mean ± SD	Minimum	Maximum
**Age (years)**	46 ± 7	33	59
**Height (m)**	1.70 ± 0.1	1.48	1.82
**Weight (kg)**	66.0 ± 14.9	49.0	99.3

**Table 2:** Spatiotemporal and kinematic gait parameters computed over the 3-D body joint data.

Gait Parameter	Definition
*Stride duration*	Duration of the gait cycle or stride
*Step duration*	Duration of the left/right step, when considering a left/right gait cycle
*Stance duration*	Duration of the stance phase
*Swing duration*	Duration of the swing phase
*Single support duration*	Duration of the single-limb support phase
*Double support duration*	Duration of the double-limb support phase
*Stride length*	Distance (in the xz-plane) between the position of the left/right ankle at the beginning and end of the left/right gait cycle
*Step length*	Distance (in the xz-plane) between the position of the left/right ankle at the instant during the swing phase when the ankle distance is minimum, and at the instant corresponding to the end of the left/right gait cycle
*Step width*	Minimum value of the distance between ankles (in the xz-plane) during the swing phase
*Gait speed*	Mean value of the trunk velocity (mean of velocity of the head, neck, spine shoulder, spine middle, and spine base joints)
*Gait speed variability*	Standard deviation of the trunk velocity
*Foot swing velocity*	Maximum value of the left/right ankle joint velocity during the swing phase, when considering a left/right gait cycle
*Arm swing velocity*	Maximum value of the left/right hand velocity (mean of velocity of the left/right wrist and hand joints) during the stance phase, when considering a left/right gait cycle
*TBCM sway x/y-component*	Measure of the body sway computed as the mean distance (in the x/y-axis) of the total body center of mass (TBCM), in relation to the RGB-D sensor’s coordinate system, for all gait cycle frames. For each frame, TBCM’s position is the mean position of all body segments’ CM, which was obtained according to [28] using the parameters indicated by Dempster in [29].
*Spine shoulder angle*	Mean value of the angle at spine shoulder (defined by head, spine shoulder, and spine base joints), considering the whole gait cycle
*Spine middle angle*	Mean value of the angle at spine middle (defined by head, spine middle, and spine base joints), considering the whole gait cycle
*Maximum elbow angle*	Maximum value of angle at the left/right elbow (defined by left/right shoulder, elbow and wrist joints) during the stance phase, when considering a left/right gait cycle
*Minimum elbow angle*	Minimum value of angle at the left/right elbow during the stance phase, when considering a left/right gait cycle
*Maximum knee angle*	Maximum value of angle at the left/right knee (defined by left/right hip, knee, and ankle joints) during the stance phase, when considering a left/right gait cycle
*Minimum knee angle*	Minimum value of angle at the left/right knee during the swing phase, when considering a left/right gait cycle
*Hip angle range*	Difference between the maximum and minimum value for the left/right hip angle (defined by left/right knee joint, hip joint, and point with knee y-coordinate and hip x- and z-coordinates) during the stance and swing phases respectively, when considering a left/right gait cycle
*Ankle angle range*	Difference between the maximum and minimum value for left/right ankle (defined by left/right knee, ankle, and foot joints) during the swing and stance phases respectively, when considering a left/right gait cycle

**Table 3:** Mean and standard deviation (SD) values obtained with Kinect and Qualisys, for each gait parameter. The Bland Altman’s mean difference (Mean diff) and 95% limits of agreement (LoA), as well as the Pearson’s correlation coefficient (*r*) and concordance correlation coefficient (*r_c_*), are also included. The ˜ and ˆ symbols indicate fixed and proportional bias, respectively. The * symbol indicates a significant *r* value (*p*-value ≤ 0.05).

Gait Parameter	Mean ± SD		Mean Diff	95% LoA	*r* (95% CI)	*r_c_* (95% CI)
	Kinect	Qualisys				
*Stride duration (s)*	1.284 ± 0.202	1.289 ± 0.206	−0.005	−0.053 to 0.042	0.99 * (0.97 to 1.00)	0.99 (0.97 to 1.00)
*Step duration (s)*	0.644 ± 0.111	0.651 ± 0.112	−0.007	−0.050 to 0.035	0.98 * (0.92 to 1.00)	0.98 (0.93 to 0.99)
*Stance duration (s)*	0.838 ± 0.143	0.890 ± 0.167	−0.052 ^~^	−0.126 to 0.022	0.98 * (0.92 to 1.00)	0.92 (0.78 to 0.97)
*Swing duration (s)*	0.446 ± 0.072	0.399 ± 0.054	0.047 ^~^	−0.021 to 0.115	0.89 * (0.59 to 0.97)	0.68 (0.31 to 0.87)
*Single support duration (s)*	0.885 ± 0.126	0.789 ± 0.093	0.096 ^~^	−0.051 to 0.242	0.81 * (0.37 to 0.95)	0.57 (0.17 to 0.81)
*Double support duration (s)*	0.399 ± 0.104	0.500 ± 0.149	−0.101 ^~^	−0.258 to 0.056	0.86 * (0.50 to 0.97)	0.62 (0.23 to 0.84)
*Stride length (cm)*	94.2 ± 14.3	94.0 ± 14.1	0.2	−1.6 to 2.1	1.00 * (0.99 to 1.00)	1.00 (0.99 to 1.00)
*Step length (cm)*	43.6 ± 6.2	44.3 ± 5.4	−0.7	−5.3 to 3.8	0.93 * (0.72 to 0.98)	0.92 (0.73 to 0.98)
*Step width (cm)*	14.8 ± 2.8	14.6 ± 3.1	0.2	−1.7 to 2.1	0.95 * (0.81 to 0.99)	0.95 (0.82 to 0.99)
*Gait speed (m/s)*	0.788 ± 0.200	0.773 ± 0.200	0.015 ^~^	0.001 to 0.029	1.00 * (1.00 to 1.00)	1.00 (0.99 to 1.00)
*Gait speed variability (m/s)*	0.118 ± 0.027	0.113 ± 0.027	0.005	−0.014 to 0.025	0.93 * (0.74 to 0.98)	0.92 (0.74 to 0.98)
*Foot swing velocity (m/s)*	3.019 ± 0.479	2.934 ± 0.621	0.085 ^^^	−0.323 to 0.493	0.96 * (0.84 to 0.99)	0.93 (0.75 to 0.98)
*Arm swing velocity (m/s)*	1.613 ± 0.387	1.608 ± 0.404	0.005	−0.109 to 0.119	0.99 * (0.96 to 1.00)	0.99 (0.96 to 1.00)
*TBCM ^a^ sway x-component (mm^2^)*	0.471 ± 0.275	0.442 ± 0.304	0.029	−0.172 to 0.230	0.94 * (0.77 to 0.99)	0.94 (0.79 to 0.98)
*TBCM ^a^ sway y-component (mm^2^)*	0.101 ± 0.053	0.146 ± 0.089	−0.044 ^~,^^	−0.122 to 0.033	0.97 * (0.87 to 0.99)	0.73 (0.37 to 0.90)
*Spine shoulder angle (deg.)*	173 ± 4	170 ± 6	3 ^~,^^	−3 to 9	0.90 * (0.62 to 0.98)	0.69 (0.30 to 0.88)
*Spine middle angle (deg.)*	176 ± 2	162 ± 8	14 ^~,^^	−5 to 33	−0.51 (−0.86 to 0.18)	0.00 (0.00 to 0.00)
*Maximum elbow angle (deg.)*	155 ± 6	155 ± 4	0	−9 to 9	0.67 * (0.06 to 0.91)	0.60 (0.04 to 0.87)
*Minimum elbow angle (deg.)*	137 ± 10	134 ± 9	2	−7 to 11	0.90 * (0.62 to 0.98)	0.88 (0.62 to 0.97)
*Maximum knee angle (deg.)*	174 ± 2	174 ± 3	0	−8 to 8	−0.21 (−0.74 to 0.48)	0.00 (0.00 to 0.00)
*Minimum knee angle (deg.)*	130 ± 4	123 ± 7	8 ^~,^^	−2 to 17	0.76 * (0.26 to 0.94)	0.34 (0.00 to 0.62)
*Hip angle range (deg.)*	14 ± 4	14 ± 5	0	−11 to 10	0.30 (−0.40 to 0.78)	0.30 (−0.33 to 0.75)
*Ankle angle range (deg.)*	29 ± 7	32 ± 12	−2	−24 to 20	0.39 (−0.32 to 0.82)	0.35 (−0.28 to 0.77)

^a^ TBCM stands for total body center of mass.

## Supplementary Materials

The following are available online at https://www.mdpi.com/1424-8220/19/22/4929/s1, Table S1. Results obtained when filtering the ankle distance measure using a moving average filter with a window of size NF1, and estimating the heel strike instants using a window of size ND1 (Kinect data), for different window sizes. The results corresponding to the chosen window size pair are underlined; Table S2. Results obtained when filtering the ankle velocity measures using a moving average filter with a window of size NF2, and identifying the side of the heel strikes using a window of size ND2 (Kinect data), for different window sizes. The results corresponding to the chosen window size pair are underlined; Table S3. Results obtained when filtering the shank angle measures using a moving average filter with a window of size NF3 (Kinect data), for different window sizes. The results corresponding to the chosen window size are underlined; Table S4. Butterworth filter’s order and cut-off frequency values used for computing gait parameters from Kinect data; Table S5. Mean absolute error between Kinect and Qualisys for the spatiotemporal gait parameters, without filtering and when varying the Butterworth filter’s order between 2 and 6 (even integer values), and cut-off frequency between 1 and 9 Hz (integer values). The result corresponding to the chosen value pair for each gait parameter is underlined; Table S6. Mean absolute error between Kinect and Qualisys for the kinematic gait parameters, without filtering and when varying the Butterworth filter’s order between 2 and 6 (even integer values), and cut-off frequency between 1 and 9 Hz (integer values). The result corresponding to the chosen value pair for each gait parameter is underlined.
