# Supplementary material for: Validation of a Single RGB-D Camera for Gait Assessment of Polyneuropathy Patients
Source: Sensors (Basel). 2019 Nov 12;19(22):4929. doi: 10.3390/s19224929 (PMC6891607; doi:10.3390/s19224929)
Supplement: Supplementary file 1 [file sensors-19-04929-s001.pdf]

Validation of a Single RGB-D Camera for Gait Assessment of Polyneuropathy Patients

Maria do Carmo Vilas-Boas*, Ana Patrícia Rocha, Hugo Miguel Pereira Choupina, Márcio Neves Cardoso, José Maria Fernandes, Teresa Coelho, João Paulo Silva Cunha

Supplementary Data

*jpcunha@fe.up.pt

Gait cycle detection

Window size choice

Table S1. **Results obtained when filtering the ankle distance measure using a moving average filter with a window of size NF1, and estimating the heel strike instants using a window of size ND1 (Kinect data), for different window sizes. The results corresponding to the chosen window size pair are underlined.**

NF1	ND1	Precision (%)	Sensitivity (%)	Absolute Error (ms)
**1**	**3**	50.9	96.0	51.3 ± 35.1
**1**	**5**	89.8	69.8	61.6 ± 40.4
**1**	**7**	95.1	48.6	61.0 ± 40.2
**1**	**9**	96.0	42.0	58.6 ± 37.4
**3**	**3**	81.9	94.4	48.2 ± 35.4
**3**	**5**	93.6	89.8	51.6 ± 37.8
**3**	**7**	95.9	86.0	50.7 ± 36.7
**3**	**9**	97.4	82.0	50.3 ± 36.7
**5**	**3**	90.8	95.4	42.9 ± 38.7
**5**	**5**	96.7	91.6	41.6 ± 36.3
**5**	**7**	97.8	91.0	41.2 ± 35.9
**5**	**9**	97.9	90.0	41.1 ± 35.8
**7**	**3**	93.6	95.1	40.2 ± 38.2
**7**	**5**	96.7	94.1	39.9 ± 37.6
**7**	**7**	<u>98.4</u>	<u>91.9</u>	<u>37.8 ± 33.7</u>
**7**	**9**	98.4	91.5	37.9 ± 33.8
**9**	**3**	94.4	94.4	39.1 ± 33.9
**9**	**5**	96.9	93.1	39.2 ± 33.9
**9**	**7**	97.5	92.5	38.7 ± 33.0
**9**	**9**	98.4	91.3	37.7 ± 30.9

Table S2. Results obtained when filtering the ankle velocity measures using a moving average filter with a window of size NF2, and identifying the side of the heel strikes using a window of size ND2 (Kinect data), for different window sizes. The results corresponding to the chosen window size pair are underlined.

NF2	ND2	Precision (%)			Sensitivity (%)			Mean (Precision & Sensitivity) (%)
		Left	Right	Mean	Left	Right	Mean	
**1**	**3**	76.4	73.9	75.1	73.6	77.3	75.4	75.3
**1**	**5**	84.4	87.2	85.8	87.8	84.4	86.1	86.0
**1**	**7**	95.1	94.6	94.8	94.5	95.2	94.8	94.8
**1**	**9**	97.4	97.1	97.3	97.1	97.4	97.3	97.3
**3**	**3**	86.7	86.0	86.3	85.8	87.1	86.5	86.4
**3**	**5**	95.7	94.6	95.2	94.8	95.8	95.3	95.2
**3**	**7**	98.4	98.1	98.2	98.0	98.4	98.2	98.2
**3**	**9**	99.0	98.4	98.7	98.4	99.0	98.7	98.7
**5**	**3**	94.3	97.0	95.7	97.1	94.2	95.6	95.7
**5**	**5**	98.0	97.4	97.7	97.7	98.1	97.9	97.8
**5**	**7**	98.4	98.7	98.5	98.7	98.4	98.5	98.5
**5**	**9**	99.0	100	99.5	100	99.0	99.5	99.5
**7**	**3**	98.0	97.5	97.7	97.4	98.1	97.7	97.7
**7**	**5**	98.7	99.0	98.9	99.0	98.7	98.9	98.9
**7**	**7**	99.3	99.4	99.4	99.3	99.4	99.4	99.4
**7**	**9**	99.4	99.7	99.5	99.7	99.4	99.5	99.5
**9**	**3**	99.0	99.4	99.2	99.3	99.0	99.2	99.2
**9**	**5**	99.7	99.7	99.7	99.7	99.7	99.7	99.7
**9**	**7**	99.7	99.7	99.7	99.7	99.7	99.7	99.7
**9**	**9**	99.3	99.4	99.4	99.3	99.4	99.4	99.4
**11**	**3**	99.3	99.4	99.4	99.3	99.4	99.4	99.4
**11**	**5**	99.4	99.7	99.5	99.7	99.4	99.5	99.5
**11**	**7**	99.4	99.7	99.5	99.7	99.4	99.5	99.5
**11**	**9**	99.4	100	99.7	100	99.4	99.7	99.7
**13**	**3**	99.4	99.7	99.5	99.7	99.4	99.5	99.5
**13**	**5**	99.7	99.7	99.7	99.7	99.7	99.7	99.7
**13**	**7**	99.7	100	99.8	100	99.7	99.8	99.8
**13**	**9**	<u>100</u>	<u>100</u>	<u>100</u>	<u>100</u>	<u>100</u>	<u>100</u>	<u>100</u>
**15**	**3**	99.4	99.7	99.5	99.7	99.4	99.5	99.5
**15**	**5**	99.7	99.7	99.7	99.7	99.7	99.7	99.7
**15**	**7**	99.7	100	99.8	100	99.7	99.8	99.8
**15**	**9**	100	100	100	100	100	100	100

Table S3. Results obtained when filtering the shank angle measures using a moving average filter with a window of size NF3 (Kinect data), for different window sizes. The results corresponding to the chosen window size are underlined.

NF3	Sensitivity (%)	Absolute Error (ms)			
		Stance duration	Swing duration	Single support duration	Double support duration
**1**	98.5	63.7 ± 53.8	63.7 ± 52.2	108.9 ± 74.8	108.6 ± 85.6
**3**	98.1	58.5 ± 50.9	60.0 ± 50.1	103.0 ± 70.9	102.0 ± 79.4
**5**	98.1	58.3 ± 50.6	59.9 ± 49.0	103.0 ± 69.7	102.1 ± 78.0
**7**	97.9	56.3 ± 49.2	57.6 ± 45.9	101.1 ± 65.8	99.9 ± 77.2
**9**	<u>98.1</u>	<u>56.8 ± 49.6</u>	<u>58.1 ± 45.8</u>	<u>101.0 ± 66.0</u>	<u>100.0 ± 78.7</u>
**11**	98.1	60.5 ± 49.1	61.5 ± 46.3	111.5 ± 66.5	109.6 ± 78.5

Gait parameter computation

Butterworth filter parameter choice (Kinect)

Table S4. Butterworth filter's order and cut-off frequency values used for computing gait parameters from Kinect data.

<table>
<tr><th>Gait parameter</th><th>Order</th><th>Cut-off frequency (Hz)</th></tr>
<tr><td>Stride length</td><td colspan="2" rowspan="3">Not filtered</td></tr>
<tr><td>Step length</td></tr>
<tr><td>Step width</td></tr>
<tr><td>Gait speed</td><td rowspan="4">6</td><td rowspan="3">4</td></tr>
<tr><td>Gait speed variability</td></tr>
<tr><td>Foot swing velocity</td></tr>
<tr><td>Arm swing velocity</td><td>3</td></tr>
<tr><td>TBCM[a] sway x-component</td><td>2</td><td>1</td></tr>
<tr><td>TBCM[a] sway y-component</td><td colspan="2">Not filtered</td></tr>
<tr><td>Spine shoulder angle</td><td colspan="2" rowspan="2">Not filtered</td></tr>
<tr><td>Spine middle angle</td></tr>
<tr><td>Elbow angle maximum</td><td>2</td><td>1</td></tr>
<tr><td>Elbow angle minimum</td><td colspan="2">Not filtered</td></tr>
<tr><td>Knee angle maximum</td><td>6</td><td>3</td></tr>
<tr><td>Knee angle minimum</td><td colspan="2">Not filtered</td></tr>
<tr><td>Hip angle range</td><td rowspan="2">6</td><td>3</td></tr>
<tr><td>Ankle angle range</td><td>1</td></tr>
</table>

[a]TBCM stands for total body center of mass.

Table S5. Mean absolute error between Kinect and Qualisys for the spatiotemporal gait parameters, without filtering and when varying the Butterworth filter's order between 2 and 6 (even integer values), and cut-off frequency between 1 and 9 Hz (integer values). The result corresponding to the chosen value pair for each gait parameter is underlined.

Order	Cut-off frequency (Hz)	Stride length (cm)	Step length (cm)	Step width (cm)	Gait speed (m/s)	Gait speed variability (m/s)	Foot swing velocity (m/s)	Arm swing velocity (m/s)	Total body center of mass sway (mm^2)	
									x-component	y-component
**No filter**		1.53	2.78	1.02	0.014	0.073	0.983	0.285	0.219	0.044
**2**	**1**	1.53	3.37	10.70	0.019	0.037	0.628	0.219	0.179	0.099
**2**	**2**	1.53	2.88	5.49	0.015	0.015	0.339	0.125	0.200	0.069
**2**	**3**	1.53	2.72	3.31	0.014	0.011	0.247	0.104	0.209	0.057
**2**	**4**	1.53	2.71	2.22	0.014	0.011	0.260	0.105	0.214	0.052
**2**	**5**	1.53	2.70	1.61	0.014	0.014	0.330	0.116	0.216	0.049
**2**	**6**	1.53	2.70	1.28	0.014	0.021	0.421	0.135	0.217	0.047
**2**	**7**	1.53	2.67	1.10	0.014	0.031	0.530	0.162	0.218	0.046
**2**	**8**	1.53	2.69	1.02	0.014	0.044	0.662	0.196	0.219	0.045
**2**	**9**	1.53	2.74	1.00	0.014	0.060	0.832	0.242	0.219	0.044
**4**	**1**	1.53	4.89	12.6	0.016	0.045	0.697	0.219	0.195	0.107
**4**	**2**	1.53	3.17	5.54	0.013	0.013	0.369	0.107	0.213	0.064
**4**	**3**	1.53	2.88	2.82	0.013	0.011	0.237	0.094	0.217	0.051
**4**	**4**	1.53	2.72	1.78	0.013	0.011	0.223	0.098	0.218	0.047
**4**	**5**	1.53	2.77	1.33	0.013	0.012	0.263	0.106	0.218	0.046
**4**	**6**	1.53	2.70	1.12	0.014	0.013	0.325	0.119	0.218	0.045
**4**	**7**	1.53	2.66	1.04	0.014	0.017	0.405	0.135	0.219	0.045
**4**	**8**	1.53	2.69	1.00	0.014	0.027	0.494	0.157	0.219	0.045
**4**	**9**	1.53	2.76	0.99	0.014	0.041	0.596	0.183	0.219	0.045
**6**	**1**	1.53	6.91	13.49	0.016	0.051	0.693	0.218	0.200	0.111
**6**	**2**	1.53	3.36	5.75	0.013	0.013	0.387	0.103	0.215	0.061
**6**	**3**	1.53	3.00	2.71	0.013	0.011	0.233	0.091	0.217	0.049
**6**	**4**	1.53	2.75	1.68	0.013	0.011	0.215	0.096	0.218	0.046
**6**	**5**	1.53	2.72	1.29	0.013	0.012	0.248	0.104	0.218	0.046
**6**	**6**	1.53	2.75	1.11	0.014	0.012	0.299	0.115	0.218	0.045
**6**	**7**	1.53	2.68	1.03	0.014	0.014	0.368	0.129	0.219	0.045
**6**	**8**	1.53	2.71	1.00	0.014	0.021	0.450	0.148	0.219	0.045
**6**	**9**	1.53	2.71	0.99	0.014	0.034	0.539	0.172	0.219	0.045

Table S6. Mean absolute error between Kinect and Qualisys for the kinematic gait parameters, without filtering and when varying the Butterworth filter's order between 2 and 6 (even integer values), and cut-off frequency between 1 and 9 Hz (integer values). The result corresponding to the chosen value pair for each gait parameter is underlined.

Order	Cut-off frequency (Hz)	Mean angle (deg.)		Elbow angle (deg.)		Knee angle (deg.)		Angle range (deg.)	
		Spine shoulder	Spine middle	Maximum	Minimum	Maximum	Minimum	Hip	Ankle
No filter		<u>4.08</u>	<u>14.69</u>	5.74	<u>5.67</u>	5.04	<u>7.54</u>	5.45	41.22
2	1	4.08	14.69	<u>5.03</u>	7.71	5.14	19.79	7.62	12.62
2	2	4.08	14.69	5.04	6.66	4.79	12.72	4.53	20.21
2	3	4.08	14.69	5.06	6.33	4.81	10.32	4.25	25.38
2	4	4.08	14.69	5.09	6.16	4.85	9.32	4.50	29.27
2	5	4.08	14.69	5.15	6.05	4.88	8.81	4.74	32.63
2	6	4.08	14.69	5.21	5.97	4.92	8.47	4.95	35.66
2	7	4.08	14.69	5.28	5.90	4.95	8.22	5.12	38.37
2	8	4.08	14.69	5.37	5.82	4.99	7.99	5.26	40.82
2	9	4.08	14.69	5.53	5.72	5.04	7.74	5.39	42.77
4	1	4.08	14.69	5.11	7.83	5.10	22.35	9.88	11.84
4	2	4.07	14.69	5.08	6.55	4.81	11.68	4.76	19.57
4	3	4.08	14.69	5.06	6.26	4.79	8.83	4.23	25.01
4	4	4.08	14.69	5.08	6.15	4.82	8.43	4.62	29.08
4	5	4.08	14.69	5.11	6.07	4.87	8.41	4.87	32.21
4	6	4.08	14.69	5.16	6.02	4.91	8.35	5.02	34.92
4	7	4.08	14.69	5.22	5.97	4.94	8.26	5.11	37.53
4	8	4.08	14.69	5.29	5.92	4.96	8.14	5.19	39.97
4	9	4.08	14.69	5.35	5.85	4.99	8.00	5.28	41.77
6	1	4.08	14.69	5.15	7.93	5.13	23.83	10.86	<u>11.45</u>
6	2	4.07	14.69	5.11	6.50	4.87	11.59	4.91	19.36
6	3	4.08	14.69	5.06	6.25	<u>4.78</u>	8.23	<u>4.22</u>	24.64
6	4	4.08	14.69	5.07	6.14	4.80	8.16	4.66	29.00
6	5	4.08	14.69	5.11	6.07	4.86	8.40	4.91	32.35
6	6	4.08	14.69	5.14	6.03	4.91	8.39	5.03	34.87
6	7	4.08	14.69	5.19	5.99	4.94	8.29	5.11	37.26
6	8	4.08	14.69	5.26	5.94	4.97	8.19	5.17	39.68
6	9	4.08	14.69	5.33	5.89	4.97	8.06	5.24	41.63
